# Nematode parasites of four species of *Carangoides* (Osteichthyes: Carangidae) in New Caledonian waters, with a description of *Philometra dispar* n. sp. (Philometridae)

**DOI:** 10.1051/parasite/2016049

**Published:** 2016-09-12

**Authors:** František Moravec, Delphine Gey, Jean-Lou Justine

**Affiliations:** 1 Institute of Parasitology, Biology Centre of the Czech Academy of Sciences Branišovská 31 370 05 České Budějovice Czech Republic; 2 Service de Systématique moléculaire, UMS 2700 CNRS, Muséum National d’Histoire Naturelle, Sorbonne Universités CP 26 43 rue Cuvier 75231 Paris cedex 05 France; 3 ISYEB, Institut Systématique, Évolution, Biodiversité, UMR7205 CNRS, EPHE, MNHN, UPMC, Muséum National d’Histoire Naturelle, Sorbonne Universités CP51 57 rue Cuvier 75231 Paris cedex 05 France

**Keywords:** Parasitic nematode, New species, Marine fish, New Caledonia, South Pacific

## Abstract

Parasitological examination of marine perciform fishes belonging to four species of *Carangoides*, i.e. *C*. *chrysophrys*, *C*. *dinema*, *C*. *fulvoguttatus* and *C*. *hedlandensis* (Carangidae), from off New Caledonia revealed the presence of nematodes. The identification of carangids was confirmed by barcoding of the COI gene. The eight nematode species found were: Capillariidae gen. sp. (females), *Cucullanus bulbosus* (Lane, 1916) (male and females), *Hysterothylacium* sp. third-stage larvae, *Raphidascaris* (*Ichthyascaris*) sp. (female and larvae), *Terranova* sp. third-stage larvae, *Philometra dispar* n. sp. (male), *Camallanus carangis* Olsen, 1954 (females) and *Johnstonmawsonia* sp. (female). The new species *P*. *dispar* from the abdominal cavity of *C*. *dinema* is mainly characterised by the body length (5.14 mm), the lengths of markedly unequal spicules (163 and 96 μm) and gubernaculum (102 μm long) provided with a dorsal protuberance and a small, reflexed dorsal barb on its posterior portion. The finding of *C*. *bulbosus* represents the first record of this parasite a century after its discovery; the first study of this species by scanning electron microscopy (SEM) enabled detailed redescription. The finding of *Johnstonmawsonia* sp. in *C*. *fulvoguttatus* is the first record of a rhabdochonid nematode from a host belonging to the Carangidae family. *Johnstonmawsonia africana* Moravec & Puylaert, 1970 and *J*. *campanae* Puylaert, 1973 are transferred to *Prosungulonema* Roytman, 1963 as *P*. *africanum* (Moravec & Puylaert, 1970) comb. n. and *P*. *campanae* (Puylaert, 1973) n. comb.

## Abstract (fr)

L’examen parasitologique de poissons perciformes marins appartenant à quatre espèces de *Carangoides*, *C. chrysophrys*, *C. dinema*, *C. fulvoguttatus* et *C. hedlandensis* (Carangidae) de Nouvelle-Calédonie a révélé la présence de nématodes. L’identification des carangidés a été confirmée par barcoding du gène COI. Les huit espèces de nématodes trouvées étaient: Capillariidae gen. sp. (femelles), *Cucullanus bulbosus* (Lane, 1916) (mâles et femelles), *Hysterothylacium* sp. (larves de troisième stade), *Raphidascaris (Ichthyascaris)* sp. (femelles et larves), *Terranova* sp. (larves de troisième stade), *Philometra dispar* n. sp. (mâle), *Camallanus carangis* Olsen, 1954 (femelles) et *Johnstonmawsonia* sp. (femelle). La nouvelle espèce *P. dispar*, de la cavité abdominale de *C. dinema*, se caractérise principalement par la longueur du corps (5.14 mm), les longueurs des spicules sensiblement inégales (163 et 96 μm) et un gubernaculum (102 μm de long) montrant une protubérance dorsale et un petit ardillon dorsal orienté vers l’arrière sur sa partie postérieure. La trouvaille de *C. bulbosus* représente la première mention de ce parasite, un siècle après sa découverte; la première étude de cette espèce par MEB a permis une redescription détaillée de l’espèce. La découverte de *Johnstonmawsonia* sp. chez *C. fulvoguttatus* est la première mention d’un nématode Rhabdochonidae chez un hôte appartenant à la famille Carangidae. *Johnstonmawsonia africana* Moravec & Puylaert, 1970 et *J. campanae* Puylaert, 1973 sont transférés vers *Prosungulonema* Roytman, 1963 comme *P. africanum* (Moravec & Puylaert, 1970) n. comb. et *P. campanae* (Puylaert, 1973) n. comb.

## Introduction

*Carangoides* Bleeker (Carangidae, Perciformes) is a genus comprising at present 21 species of marine fishes that inhabit the tropical and subtropical regions of the Indian, Pacific and Atlantic Oceans [14]. In 2009 and 2010, during extensive studies of the parasites of marine fishes in New Caledonian waters, specimens of four species of *Carangoides* were examined. Since no data on the parasites of *Carangoides* spp. from off New Caledonia were available, the newly obtained helminthological material has provided the first information from this zoogeographically interesting region.

Based on this material, digeneans [4, 9–12] and trypanorhynch cestodes [8] have already been recorded. Regarding the parasitic nematodes, Moravec & Justine [40] mentioned the finding of the unidentified capillariid female, Capillariidae gen. sp., from *C*. *dinema* Bleeker (erroneously reported as *C*. *oblongus* (Cuvier) – see Bray & Justine [12]), and Shamsi et al. [64] recorded four ascaridoid larval types, *Anisakis* type I, *Raphidascaris* type and *Terranova* types I and II, in five *Carangoides* spp. Results of the evaluation of nematodes collected from four species of congeneric hosts from off New Caledonia are presented herein.

## Materials and methods

### Fish and their identification

Fish were purchased from the fish market in Nouméa, New Caledonia. Most fishes from the fishmarket were taken with mackerel nets within a few miles off Nouméa and were very fresh. All carangids were relatively young specimens, far from the maximum lengths reported for these species [67]. The following fish species were examined: *Carangoides chrysophrys* (Cuvier) (*n* = 3), *C*. *dinema* (*n* = 7), *C*. *fulvoguttatus* (Forsskål) (*n* = 10) and *C*. *hedlandensis* (Whitley) (*n* = 2) (Table 1). Fish were identified by their morphology, and confirmation of identification, from photographs of specimens, was sought from experts in ichthyology (Ronald Fricke, Bernard Séret and Samuel Iglésias). Fish DNA was extracted from tissue samples using the NucleoSpin 96 Tissue kit (Macherey-Nagel, Düren, Germany) following the manufacturer’s instructions. Sequences were obtained by amplification and sequencing the 5′ region of the cytochrome oxidase subunit I (COI) mitochondrial gene using the primers FishF1 (5′-TCAACYAATCAYAAAATYGGCAC-3′) and FishR1 (5′-TGATTYTTYGGYCACCCRGAAGT-3′) [70]. Standard PCRs were carried out in a total volume of 20 μL, containing about 30 ng of DNA, 1 × 10× PCR buffer, 2 mM MgCl_2_, 200 μM mix dNTPs, 150 nM of each primer and 1 unit of Taq polymerase (Qiagen, Hilden, Germany). After an initial denaturation of 3 min at 95 °C, the mitochondrial DNA was amplified through 39 cycles of 15 s at 95 °C, 20 s at 48 °C and 40 s at 72 °C, with a terminal elongation for 5 min at 72 °C. PCR products were purified and sequenced in both directions on a 3730xl DNA Analyser 96-capillary sequencer (Applied Biosystems, Waltham, MA, USA). Sequences were edited using CodonCode Aligner software (CodonCode Corporation, Dedham, MA, USA), compared with the GenBank database content using BLAST and deposited in GenBank under Accession Numbers KX712506–KX712510. Species identification was confirmed using the BOLD identification engine [59] and BLAST in GenBank. The fish nomenclature adopted follows FishBase [18].

**Table 1.** Specimens of fish positive for nematodes, their characteristics and COI barcoding sequences, and nematodes found.

Species	MHNN JNC #	Date	Fork length (mm)	Weight (g)	COI sequence	Nematodes
*Carangoides chrysophrys*	JNC3212	21 July 2010	265	398	KX712510	*Camallanus carangis*
*Carangoides dinema*	JNC3184	4 June 2010	315	708	KX712509	*Raphidascaris* (*Ichthyascaris*) sp.
	JNC2880	13 March 2009	310	624	–	Capillariidae gen. sp 3
	JNC3224	26 August 2010	320	650	–	*Raphidascaris* (*Ichthyascaris*) sp.
						*Philometra dispar* n. sp.
	JNC3225	26 August 2010	305	592	–	Capillariidae gen. sp 3
						*Raphidascaris* (*Ichthyascaris*) sp.
*Carangoides fulvoguttatus*	JNC3176	28 May 2010	270	340	KX712507	*Cucullanus bulbosus*
						*Hysterothylacium* sp.
						*Raphidascaris* (*Ichthyascaris*) sp.
						*Terranova* sp.
						*Johnstonmawsonia* sp.
	JNC3180	3 June 2010	295	430	KX712508	Capillariidae gen. sp 3
						*Cucullanus bulbosus*
						*Hysterothylacium* sp.
						*Raphidascaris* (*Ichthyascaris*) sp.
*Carangoides hedlandensis*	JNC3172	27 May 2010	245	341	KX712506	*Camallanus carangis*

### Nematodes

Parasites were collected using a “wash” method [25]. The nematodes were fixed in hot 4% formalin or 70% ethanol. For light microscopic examination, they were cleared with glycerine. Drawings were made with the aid of a Zeiss drawing attachment. Specimens used for scanning electron microscopy were postfixed in 1% osmium tetroxide (in phosphate buffer), dehydrated through a graded acetone series, critical-point-dried and sputter-coated with gold; they were examined using a JEOL JSM-7401F scanning electron microscope at an accelerating voltage of 4 kV (GB low mode). All measurements are in micrometres unless indicated otherwise. The classification system of the Ascaridoidea adopted follows Keys to the Nematode Parasites of Vertebrates [1, 19].

## Molecular identification of fish

*Carangoides chrysophrys.* The single sequence (GenBank KX712510) obtained from fish JNC3212 was 99.23–100% identical to sequences of *C. chrysophrys* included in BOLD and/or GenBank.

*Carangoides dinema.* The single sequence (GenBank KX712509) obtained from fish JNC3184 was 99.85–100% identical to sequences of *C. dinema* included in BOLD and/or GenBank.

*Carangoides fulvoguttatus.* The two sequences (GenBank KX712507, KX712508) obtained from fish JNC3176 and JNC3180 were identical. They were 99.65–100% identical to sequences of *C. fulvoguttatus* included in BOLD and/or GenBank.

*Carangoides hedlandensis.* The single sequence (GenBank KX712506) obtained from fish JNC3172 was 99.53–100% identical to sequences of *C. hedlandensis* included in BOLD and/or GenBank.

In all these cases, we consider that our morphological identifications were confirmed by high similarity (>99%) or identity (100%) to sequences registered under the same taxon name in databases.

## Capillariidae gen. sp. 3 of Moravec & Justine, 2010 (Fig. 1)

Fam. Capillariidae Railliet, 1915

**Figure 1.**
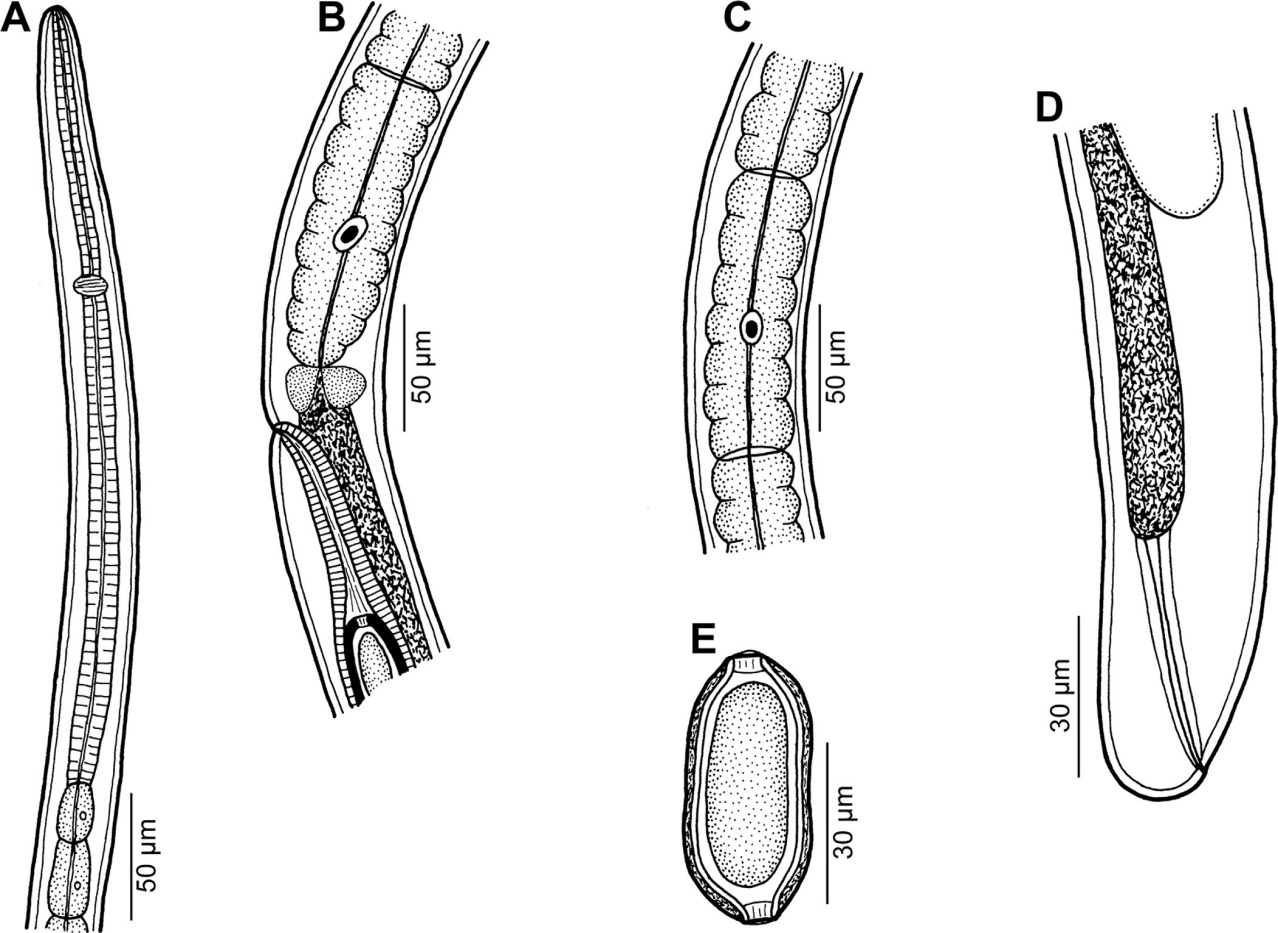
Capillariidae gen. sp., gravid female from *Carangoides dinema*. A: Anterior end of body. B: Region of vulva, lateral view. C: Stichocyte from middle part of stichosome. D: Posterior end of body, lateral view. E: Egg.

Hosts: Shadow trevally *Carangoides dinema* and yellowspotted trevally *C*. *fulvoguttatus* (both Carangidae, Perciformes).

Site of infection: Digestive tract.

Locality: Fish market, Nouméa, New Caledonia (JNC2880, collected 13 March 2009; JNC3180, 3 June 2010; JNC3225, 26 August 2010).

Prevalence and intensity: in 2 of 7 *C*. *dinema* and in 1 of 10 *C*. *fulvoguttatus* examined; 1 nematode per fish.

Deposition of voucher specimens: Muséum National d’Histoire Naturelle, Paris (MNHN JNC2880, JNC3225, *C. dinema*; MNHN JNC3180D, *C. fulvoguttatus*).

### Description

*Female* (three gravid specimens): Medium-sized filiform nematodes. Anterior end of body narrow; cephalic papillae indistinct (Fig. 1A). Length of body 11.75–14.55 mm, maximum width 60–69. Two lateral, fairly wide bacillary bands extending along almost whole body length; their width at region of posterior end of oesophagus 18–24. Length of entire oesophagus 6.84–7.40 mm, representing 51–61% of body length. Muscular oesophagus 207–354 long (Fig. 1A). Stichosome consisting of single row of elongate stichocytes subdivided usually (mainly in its middle and posterior parts) into many (8–15) transverse annuli (Figs. 1B, 1C); nuclei of stichocytes large. Length of stichosome 6.63–7.05 mm; stichocytes approximately 50 in number. Nerve ring encircling muscular oesophagus at about its one third, 81–120 from anterior extremity (Fig. 1A). Two small wing-like cells present at oesophago-intestinal junction (Fig. 1B). Vulva located 6.92–7.53 mm from anterior end of body (at 52–61% of body length), 13–132 posterior to level of oesophago-intestinal junction (Fig. 1B); vulval lips not elevated or anterior lip slightly elevated. Vagina short, muscular. Eggs in anterior part of uterus arranged in single row, more distant eggs in two rows. Eggs oval, usually somewhat narrowed equatorially, with slightly protruding polar plugs (Fig. 1E). Egg wall appearing as two-layered; inner layer hyaline, outer layer thicker, with fine superficial net-like sculpture. Eggs including polar plugs 57–60 × 24–30, thickness of egg wall 3–4; polar plugs 6 long and 6 wide. Content of fully developed eggs uncleaved. Caudal end rounded; anus subterminal, length of tail 6–12. Rectum formed by hyaline tube 60–66 long (Fig. 1D).

*Male*: Not known.

### Remarks

Available female specimens cannot be identified to generic level, because conspecific males are absent. These nematodes are characterised mainly by a relatively short body length and muscular oesophagus, stichocytes with distinct transverse annuli, a subterminal anus and especially by the shape, structure and size of eggs. To date, three species of capillariids have been reported from marine fishes of the family Carangidae: *Pseudocapillaria carangi* (Parukhin, 1971), *P*. *decapteri* (Luo, 2001) and *Capillaria gracilis* (Bellingham, 1840) [29, 35]. Whereas *C*. *gracilis* is a parasite mainly of gadiform fishes and its record in the carangid *Trachinotus carolinus* (Linnaeus) may well be a misidentification, *P*. *carangi* and *P*. *decapteri*, both inadequately described, are reported only from members of Carangidae. Therefore, it is probable that our specimens also belong to *Pseudocapillaria* Freitas, 1959.

Parukhin [51–53] reported *P*. *carangi* from 12 species of carangid fishes (including two *Carangoides* spp.) from the western part of the Indian Ocean (Monar Bay, Arabian Sea near Oman, Gulf of Aden, Red Sea, off southeastern coast of Africa), whereas *P*. *decapteri* was recorded from the North Pacific Ocean near Japan [29]. Although specimens of the present material may belong to one of these two species (which, however, may be identical to each other), their poor original descriptions and principally the absence of a male in our material do not allow us to assign the New Caledonian specimens to a species.

The finding of one female specimen of this species, reported as Capillariidae gen. sp. 3, in New Caledonian waters was recorded by Moravec & Justine [40]; however, the host reported as *Carangoides oblongus* (Cuvier) was in fact *C*. *dinema* [12].

## *Cucullanus bulbosus* (Lane, 1916) Barreto, 1918 (Figs. 2, 3)

Syn.: *Bulbodacnitis bulbosa* Lane, 1916.

**Figure 2.**
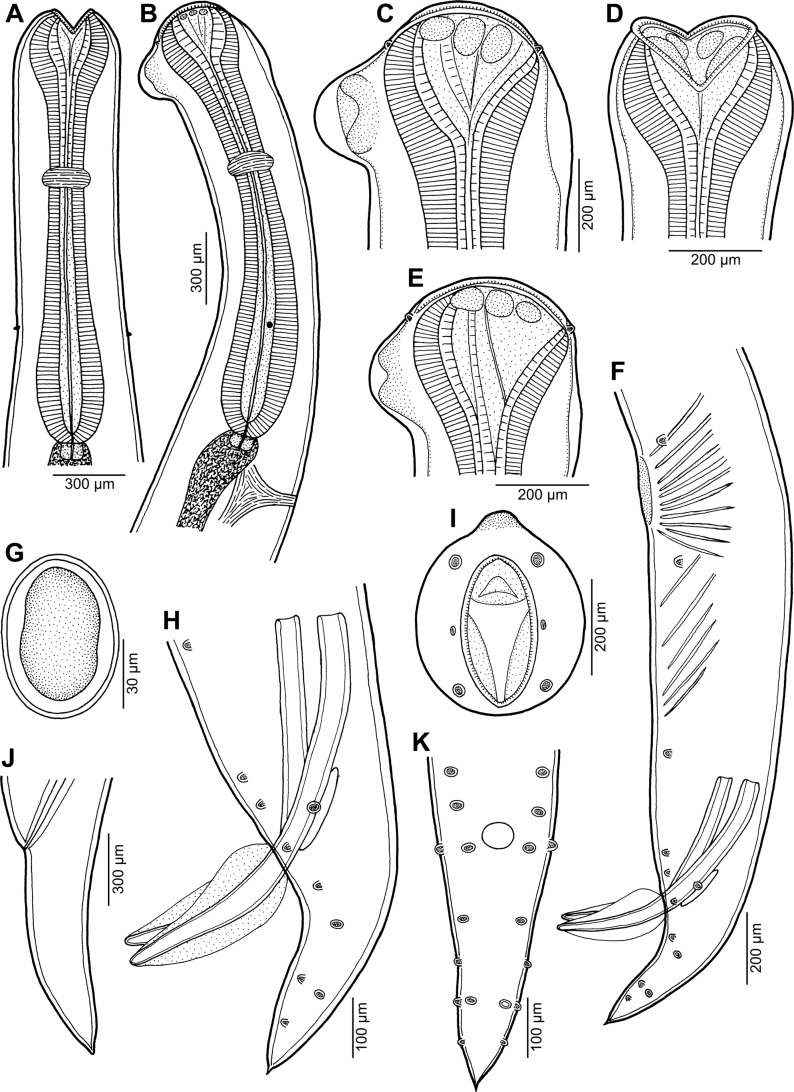
*Cucullanus bulbosus* (Lane, 1916) from *Carangoides fulvoguttatus*. A, B: Anterior end of gravid female, dorsoventral and lateral views, respectively. C, D: Cephalic end of gravid female, lateral and ventral views, respectively. E: Cephalic end of male, lateral view. F: Posterior end of male, lateral view. G: Egg. H: Caudal end of male, lateral view. I: Cephalic end of male, apical view. J: Tail of gravid female, lateral view. K: Caudal end of male, ventral view.

**Figure 3.**
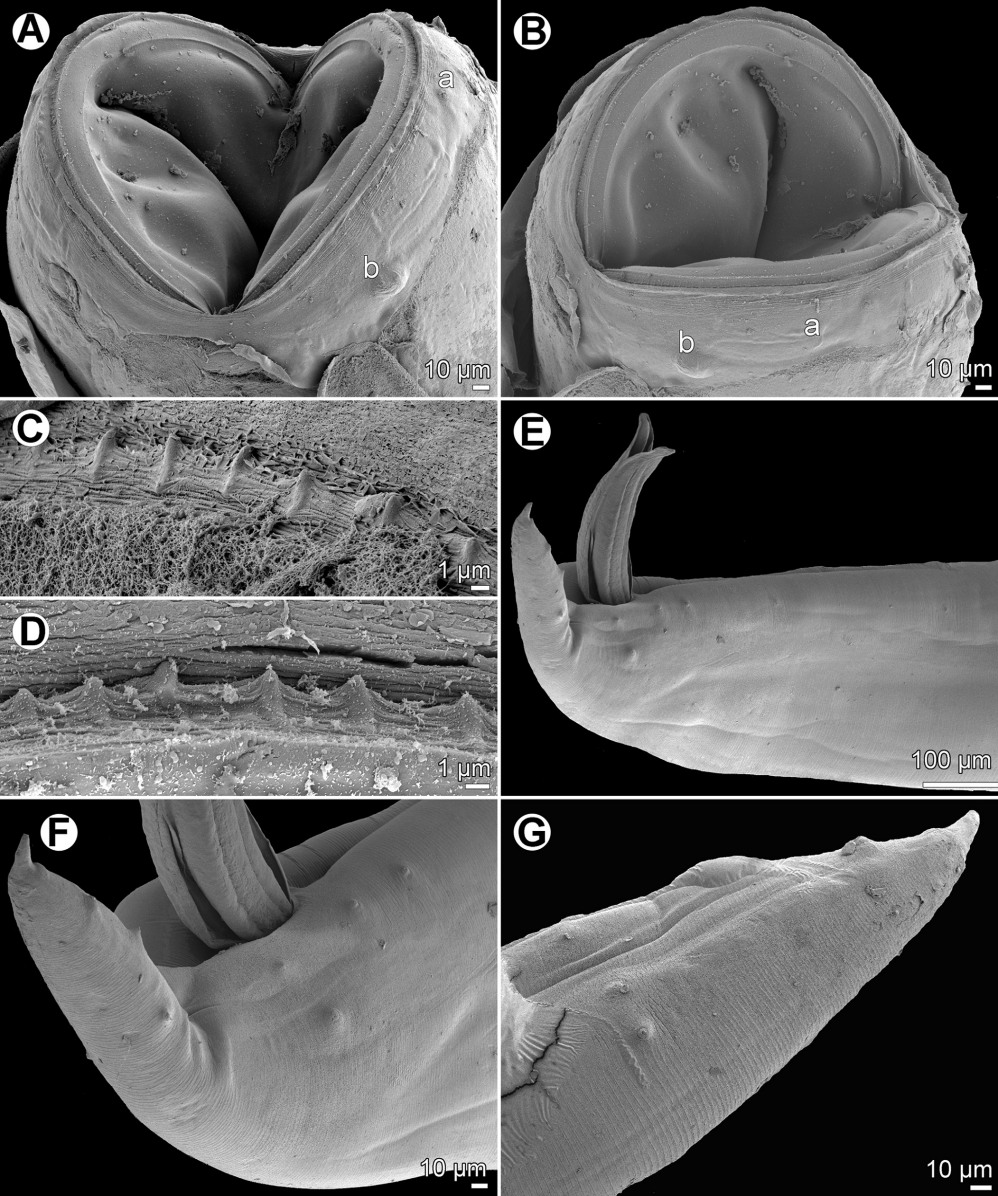
*Cucullanus bulbosus* (Lane, 1916), scanning electron micrographs. A, B: Cephalic end of nongravid female, ventral and lateral views, respectively. C, D: Peribuccal teeth of male and nongravid female, respectively. E: Posterior of male, lateral view. F: Tail of male, lateral view. G: Posterior part of male tail, lateral view. *Abbreviations*: a, amphid; b, cephalic papilla.

Fam. Cucullanidae Cobbold, 1864

Host: Yellowspotted trevally *Carangoides fulvoguttatus* (Carangidae, Perciformes).

Site of infection: Digestive tract.

Locality: Fish market, Nouméa, New Caledonia (JNC3176, collected 28 May 2010; JNC3180, collected 3 June 2010).

Prevalence and intensity: in 2 of 10 *C*. *fulvoguttatus* examined; 1 and 2 nematodes.

Deposition of voucher specimen: Muséum National d’Histoire Naturelle, Paris (MNHN JNC3176).

### Description

*General*: Medium-sized nematodes. Body whitish, elongate, with anterior end somewhat curved dorsally. Cuticle slightly transversely striated. Lateral alae absent. Cephalic end somewhat asymmetrical in lateral view, with conspicuous large dorsal hemispherical elevation at level of pseudobuccal capsule (Figs. 2B, 2C and 2E). Oral aperture dorsoventrally elongate, surrounded by raised narrow membranous ala (collarette) supported by row of minute basal teeth (Figs. 2D, 2I and 3A–3D). Four submedian cephalic papillae and pair of lateral amphids present (Figs. 2C, 2E, 2I, 3A and 3B). Oesophagus muscular, somewhat expanded at anterior end to form rather large pseudobuccal capsule (oesophastome); posterior part of oesophagus also expanded, slightly narrower than pseudobuccal capsule (Figs. 2A and 2B); cuticular lining of oesophastome consists of complex set of thickened cuticularised pieces separated by sutures (Figs. 2A–2E, 2I, 3A and 3B). Oesophagus opens into intestine through large valve. Nerve ring encircles oesophagus at distance representing 39–41% of oesophageal length. Deirids small, hooked, at short distance anterior to end of oesophagus (Figs. 2A and 2B). Postdeirids not found. Excretory pore slightly posterior to oesophago-intestinal junction (Fig. 2B). Tail conical, with sharply pointed tip.

*Male* (1 specimen): Length of body 16.19 mm, maximum width 571; width at region of oesophastome including dorsal elevation 476, at region of middle of oesophagus 381. Length of entire oesophagus 1.97 mm, representing 12% of body length; length of oesophastome 354, its width 367; minimum width of oesophagus 163; maximum width of posterior part of oesophagus 313. Distance from nerve ring to anterior extremity 726, representing 39% of oesophageal length. Deirids and excretory pore 1.63 and 2.15 mm, respectively, from anterior end of body. Posterior end of body curves ventrally. Ventral region of cloacal opening not elevated. Spicules equal, 830 long, their distal parts provided with markedly wide dorsal and ventral alae; maximum width of spicules including alae 136 (Figs. 2F, 2H, 3E and 3F). Gubernaculum well sclerotised, narrow in lateral view, 218 long. Ventral sucker and oblique muscle bands well developed (Fig. 2F); former situated 1.22 mm from cloacal aperture. Preanal papillae 5 subventral pairs; adanal papillae 1 subventral pair and 1 lateral pair; postanal papillae 3 subventral and 2 lateral pairs (Figs. 2F, 2H, 2K, 3E and 3F). Length of tail 435.

*Female* (1 gravid and 1 nongravid specimen; measurement of latter in parentheses): Length of body 18.39 (12.77) mm, maximum width 666 (490); width at region of oesophastome including dorsal elevation 585 (408), at region of middle of oesophagus 462 (286). Length of entire oesophagus 2.07 (1.74) mm, representing 11 (14)% of body length; length of oesophastome 381 (367), its width 367 (326); minimum width of oesophagus 150 (136); maximum width of posterior part of oesophagus 326 (231). Distance from nerve ring to anterior extremity 816 (707), representing 39 (41)% of oesophageal length. Deirids and excretory pore 1.84 (1.40) and 2.48 (2.11) mm, respectively, from anterior end of body. Vulva postequatorial, 13.74 (8.61) mm from anterior extremity, at 75 (67)% of body length; vulval lips elevated. Vagina directed anteriorly from vulva. Uteri opposed. Eggs numerous (eggs absent in nongravid specimens); fully developed eggs oval, thin-walled, with contents uncleaved or cleaved at most into several blastomeres (Fig. 2G); eggs 82 long, 54 wide. Length of tail 462 (422) (Fig. 2J).

### Remarks

Lane [28] described a new cucullanid species, *Bulbodacnitis bulbosa*, from the bluefin trevally *Caranx melampygus* Cuvier off Sri Lanka and established the new genus *Bulbodacnitis* to accommodate it, because he considered the presence of the dorsal hemispherical cephalic elevation in this species to be of generic importance. However, Barreto [5, 6] considered *Bulbodacnitis* Lane, 1916 a junior synonym of *Cucullanus* Müller, 1777, to which he transferred Lane’s species. Nevertheless, Smedley [66] and Simon [65] described two new species of *Bulbodacnitis* from North American salmonids, while other authors [7, 15, 58, 71] did not recognise *Bulbodacnitis* as an independent genus. Subsequently, Maggenti [31] re-erected *Bulbodacnitis* for the cucullanids with the oral aperture dorsally oblique to the longitudinal body axis (see also [21, 48]). However, Petter [54] pointed out that this feature is not found in *B*. *bulbosus*, the type species of *Bulbodacnitis*, and, consequently, she retained *Bulbodacnitis* as a synonym of *Cucullanus* and established a new genus *Truttaedacnitis* Petter, 1974 for the species with the distinctly oblique oral aperture. According to Moravec [34], *Truttaedacnitis* should be considered as a subgenus of *Cucullanus*.

The present specimens from *C*. *fulvoguttatus* correspond, more or less, to the description of *C*. *bulbosus*, both these forms were collected from carangid fishes, and *C*. *melampygus*, the type host of *C*. *bulbosus*, also occurs in New Caledonian waters [18]. Therefore, the New Caledonian nematodes undoubtedly belong to *C*. *bulbosus*.

*Cucullanus bulbosus* has not been recorded since its description by Lane [28], making the New Caledonian specimens the first finding of this species after a century. The original description of *Bulbodacnitis bulbosa* (= *C*. *bulbosus*) is relatively good (a somewhat modified description, based on the original one, was published by Baylis [7]). The present study, including the first scanning electron microscopy (SEM) examination, confirmed some previously reported morphological features in this species, showed some new characters (presence of circumoral spines and ventral oblique muscle bands in the male) and provided more exact observations of the cephalic structures and male caudal papillae. The finding of *C*. *bulbosus* in *C*. *fulvoguttatus* from off New Caledonia represents new host and geographical records.

## *Hysterothylacium* sp. (Figs. 4A–4C)

Fam. Anisakidae Railliet & Henry, 1912

**Figure 4.**
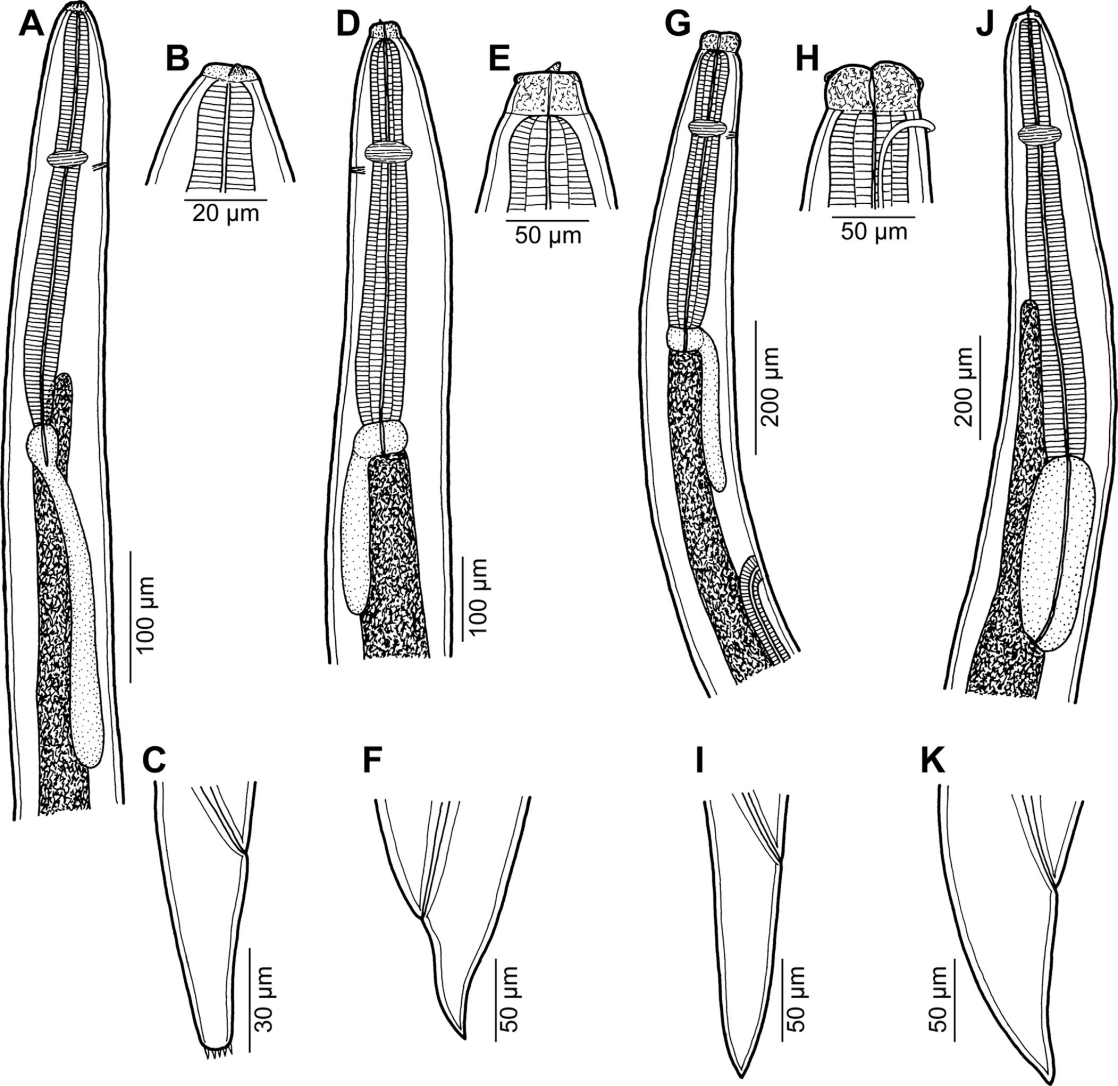
Ascaridoid larvae from *Carangoides* spp. A–C: *Hysterothylacium* sp. third-stage larva from *Carangoides fulvoguttatus* (A: anterior end of body; B: cephalic end; C: tail; all lateral views). D–F: *Raphidascaris* (*Ichthyascaris*) sp. third-stage larva from *Carangoides fulvoguttatus* (D: anterior end of body; E: cephalic end; F: tail; all lateral views). G–I: *Raphidascaris* (*Ichthyascaris*) sp. fourth-stage larva from *Carangoides fulvoguttatus* (G: anterior end of body; H: cephalic end; I: tail; all lateral views). J, K: *Terranova* sp. third-stage larva from *Carangoides fulvoguttatus* (J: anterior end of body; K: tail; both lateral views).

Host: Yellowspotted trevally *Carangoides fulvoguttatus* (Carangidae, Perciformes).

Site of infection: Digestive tract.

Locality: Fish market, Nouméa, New Caledonia (JNC3176, collected 28 May 2010; JNC3180, collected 3 June 2010)

Prevalence and intensity: in 2 of 10 *C*. *fulvoguttatus* examined; 1 nematode per fish.

Deposition of voucher specimens: Muséum National d’Histoire Naturelle, Paris (MNHN JNC3180, JNC3180D).

### Description

Third-stage larva (1 specimen): Body length 2.94 mm, maximum width 82. Cephalic end truncated, with anlagen of lips and ventral tooth (Fig. 4B). Lateral alae absent. Oesophagus 345 long, maximum width 24. Ventriculus oval, 36 long, 27 wide. Posterior ventricular appendix 267 long, 21 wide. Nerve ring and excretory pore 132 and 135, respectively, from anterior end of body. Intestine straight. Anterior intestinal caecum short, 87 long, 21 wide (Fig. 4A). Length ratio of caecum and ventricular appendix 1:3. Tail conical, 93 long, provided with several small cuticular spikes at tip (Fig. 4C); length of spines ca. 3.

### Remarks

The genus *Hysterothylacium* Ward & Magath, 1917 includes many species, which are gastro-intestinal parasites mostly of marine fishes belonging to different families and orders. To date, only three species of *Hysterothylacium* have been recorded from New Caledonian waters: *H*. *alatum* Moravec & Justine, 2015 from *Plectropomus laevis* (Lacépède) (Serranidae), *H*. *cenaticum* (Bruce & Cannon, 1989) from *Kajikia audax* (Philippi) (Istiophoridae) and *H*. *sphyraenae* Moravec & Justine, 2015 from *Sphyraena qenie* Klunzinger (Sphyraenidae) [39, 43]. In addition, unidentified larvae of *Hysterothylacium* have been reported off New Caledonia from several fish species of the Balistidae, Clupeidae, Lethrinidae, Nemipteridae, Scombridae, Serranidae, Sphyraenidae and Trichiuridae [23, 24, 64].

The life cycles and larval morphogenesis of *Hysterothylacium* spp. remain mostly unknown, making species identification of the larvae of this genus from fishes, based on morphological features, impossible. Shamsi et al. [63, 64] distinguished 14 morphotypes of larval *Hysterothylacium* spp. (types I–XIV) from marine fishes in Australian and New Caledonian waters, of which types VI, XIII and XIV were recorded from fishes from off New Caledonia [64]. However, it is necessary to note that the “sinusoidal” or “serpengenous” patterns of the intestine, reported to be characteristic of the larval types VI and XIII, were in fact the coils of the developing genital tract, as is evident from the respective microphotographs (Figs. 2A and 2C of Shamsi et al. [64]).

Based on their morphology, the present *Hysterothylacium* larvae from *C*. *fulvoguttatus* cannot be assigned to any of the congeneric larval types of Shamsi et al. [63, 64], all of which were reported from non-carangid fishes. It is not clear whether the present *Hysterothylacium* larvae may attain full maturity in *C*. *fulvoguttatus*, serving thus as the definitive host, or whether this fish is only utilised as the paratenic host. The only two species reported from carangid fishes are *H*. *chorinemi* (Parukhin, 1966), recorded from *Atule mate* (Cuvier), *Caranx sexfasciatus* Quoy & Gaimard and *Scomberoides lysan* (Forsskål) (all Carangidae) in the South China, Arabian and Red Seas and off the southeastern coast of Africa [50, 53], and *H*. *carangis* (Kalyankar, 1971), described from *Carangoides malabaricus* (Bloch & Schneider) off India [13, 26].

## *Raphidascaris* (*Ichthyascaris*) sp. (Figs. 4D–4I)

Fam. Anisakidae Railliet & Henry, 1912

Hosts: Shadow trevally *Carangoides dinema* and yellowspotted trevally *C*. *fulvoguttatus* (both Carangidae, Perciformes).

Site of infection: Digestive tract.

Locality: Fish market, Nouméa, New Caledonia (collected 28 May, 3 and 4 June and 26 August 2010).

Prevalence and intensity: *C*. *dinema*: 3 of 7 fish examined infected; 1–2 nematodes per fish. *C*. *fulvoguttatus*: 2/10; 6 and 9 nematodes.

Deposition of voucher specimens: Muséum National d’Histoire Naturelle, Paris (*C. fulvoguttatus*, JNC3176, JNC3180C; *C. dinema*, JNC3184, JNC3224, JNC3225).

### Description

*Female* (one body fragment of posterior end of gravid specimen from *C*. *dinema*): Length of body fragment 1.50 mm, maximum width 231. Tail conical, 159 long, with many small papilla-like cuticular projections at tip. Uterus containing several thin-walled, almost spherical eggs 39–42 in diameter with uncleaved content.

*Female fourth-stage larva* (two specimens from *C*. *dinema* and *C*. *fulvoguttatus*): Body length 4.45–5.70 mm, maximum width 163–245. Cephalic end with well-developed lips (Fig. 4H); lips 36–51 long. Narrow lateral alae united anteriorly close to ventrolateral lips on ventral side of body present (Fig. 4H). Oesophagus 462–680 long, maximum width 82–122. Ventriculus transverse-oval, 36–54 long and 63–95 wide. Posterior ventricular appendix 249–313 long, 33–41 wide. Nerve ring and excretory pore 190–272 and 190–272, respectively, from anterior end of body (Fig. 4G). Vulva, still covered by cuticle, located 911–1,346 from anterior extremity, i.e. at 20–24% of body length (Fig. 4G). Vagina directed posteriorly from vulva; many coils of developing genital tract present in body posterior to vulva. Tail conical, 204–218 long, pointed, without any caudal projections at tip (Fig. 4I).

*Advanced third-stage larva* (5 specimens from *C*. *fulvoguttatus*): Body length 3.79–4.58 mm, maximum width 136–163. Cephalic end without lips, bearing distinct ventral larval tooth (Fig. 4E). Lateral alae not observed. Oesophagus 408–517 long, maximum width 60–68. Ventriculus transverse-oval, 33–36 long and 51–66 wide. Posterior ventricular appendix 231–309 long, 27–45 wide. Nerve ring and excretory pore 135–165 and 135–176, respectively, from anterior end of body (Fig. 4D). Vulva and vagina absent. Body with many coils of developing genital tract. Tail conical, sharply pointed, 82–190 long (Fig. 4F).

### Remarks

All the above-mentioned forms are considered to represent one and the same species of *Raphidascaris* Railliet & Henry, 1915, which attains full maturity in *Carangoides* spp. The presence of characteristic, anteriorly united lateral alae in fourth-stage larvae shows that this currently undescribed species belongs to the subgenus *Ichthyascaris* Wu, 1949.

To date, 10 species of *Raphidascaris* (*Ichthyascaris*) are known as parasites of marine fishes [72]. Of these, five species were reported from the South Pacific Ocean in the Australian region: *R*. (*I*.) *fisheri* (Hooper, 1983), *R*. (*I*.) *gymnocraniae* (Bruce, 1990) and *R*. (*I*.) *sillagoides* (Bruce, 1990) in Australian waters and *R*. (*I*.) *etelidis* Moravec & Justine, 2012 and *R*. (*I*.) *nemipteri* Moravec & Justine, 2005 from off New Caledonia [13, 39, 41]. However, none of the *Raphidascaris* (*Ichthyascaris*) spp. has so far been described from fishes of the family Carangidae. Therefore, it can be assumed that the nematodes parasitising *Carangoides* spp. in New Caledonian waters belong to a new species.

Ascaridoid larvae designated as “*Raphidascaris* larval type” were reported from *Carangoides chrysophrys* from off New Caledonia by Shamsi et al. [64].

## *Terranova* sp. (Figs. 4J, 4K)

Fam. Anisakidae Railliet & Henry, 1912

Host: Yellowspotted trevally *Carangoides fulvoguttatus* (Carangidae, Perciformes).

Site of infection: Digestive tract.

Locality: Fish market, Nouméa, New Caledonia (collected 28 May 2010).

Prevalence and intensity: in 1 of 10 *C*. *fulvoguttatus* examined; 4 nematodes.

Deposition of voucher specimens: Muséum National d’Histoire Naturelle, Paris (MNHN JNC3176).

### Description

*Third-stage larva* (four specimens): Body length 6.61–9.02 mm, maximum width 204–286. Cephalic end truncated, with anlagen of lips and distinct ventral tooth. Oesophagus 802–952 long, maximum width 68–95. Ventriculus large, oval, 340–435 long, 109–136 wide. Nerve ring 231–286 from anterior end of body. Excretory pore just posterior to larval tooth. Anterior intestinal caecum 612–748 long, 41–68 wide (Fig. 4J). Tail conical, 109–122 long, pointed (Fig. 4K).

### Remarks

Larvae of this type, designated as *Terranova* type II, were already reported from off New Caledonia by Shamsi et al. [64], who had recorded them from fishes of the families Carangidae (including *C*. *fulvoguttatus*), Chirocentridae, Monodactylidae, Scombridae, Serranidae, Sphyraenidae and Trichiuridae.

Species of *Terranova* Leiper & Atkinson, 1914 are parasites of the digestive tract of fishes and reptiles. Many species of teleost fishes serve only as paratenic hosts of larvae, which is apparently the case of carangids.

## *Philometra dispar* n. sp. (Figs. 5, 6)

Fam. Philometridae Baylis & Daubney, 1926

**Figure 5.**
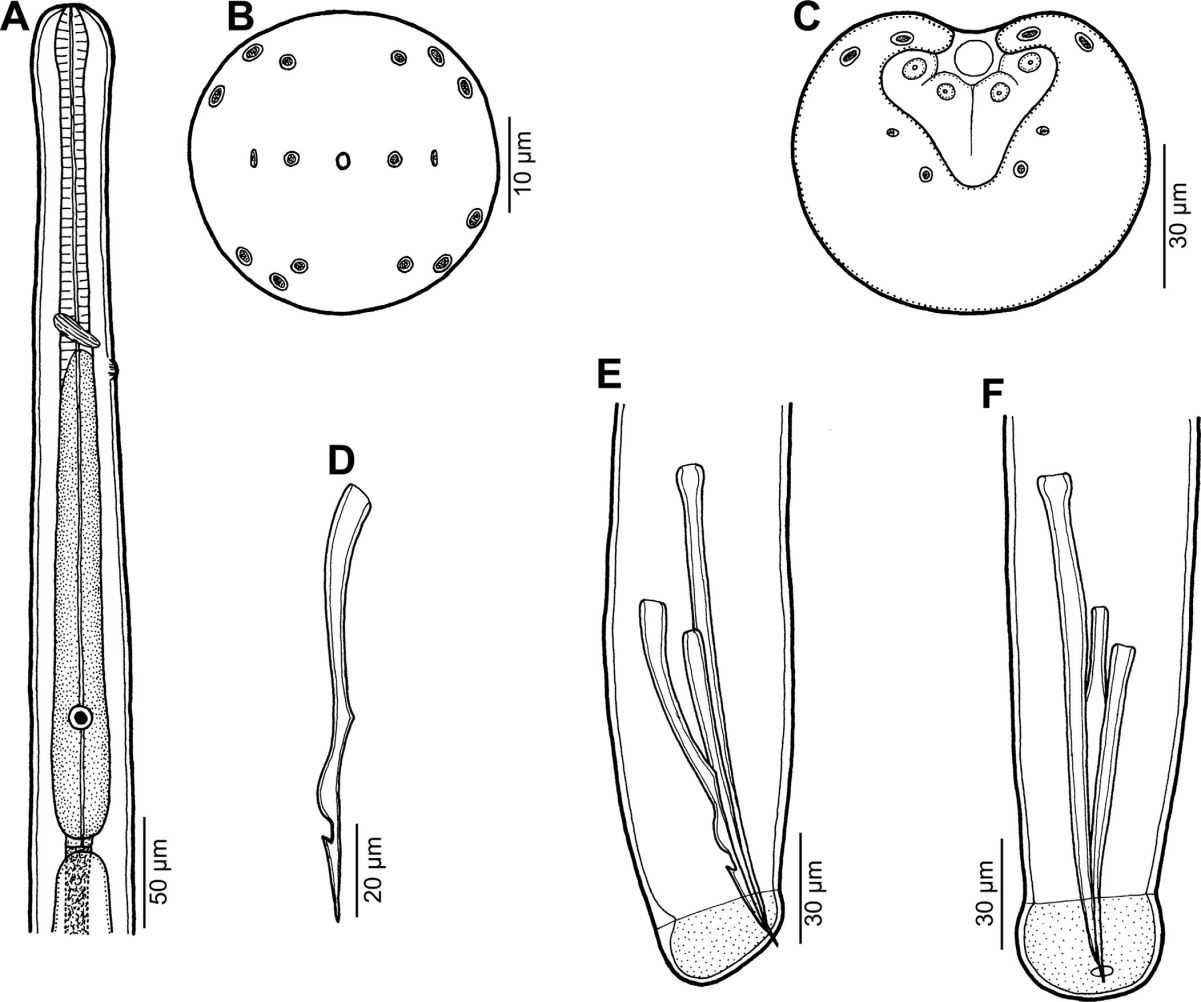
*Philometra dispar* n. sp. from *Carangoides dinema*, male. A: Anterior end of body, lateral view. B: Cephalic end, apical view. C: Caudal end, apical view. D: Gubernaculum, lateral view. E, F: Posterior end, lateral and ventral views.

**Figure 6.**
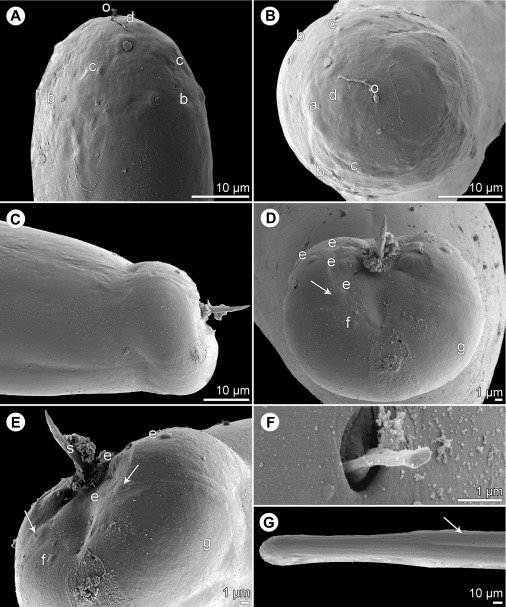
*Philometra dispar* n. sp. from *Carangoides dinema*, scanning electron micrographs of male. A, B: Cephalic end, dorsoventral and apical views, respectively. C, D: Caudal end, lateral and apical views, respectively (arrow indicates phasmid). E: Caudal end, subdorsal view (arrows indicate phasmids). F: Deirid. G: Anterior end of body, dorsoventral view (arrow indicates location of deirid). *Abbreviations*: a, amphid; b, submedian pair of cephalic papillae of external circle; c, submedian cephalic papilla of internal circle; d, lateral cephalic papilla of internal circle; e, caudal papillae in region of cloacal aperture; f, caudal papilla of last postanal pair; g, caudal mound; o, oral aperture.

urn:lsid:zoobank.org:act:C4134134-E130-48D7-AE28-EA4B5F3CC288

Type host: Shadow trevally *Carangoides dinema* (Carangidae, Perciformes); JNC3224 (see Table 1); Fork length 320 mm, weight 650 g.

Site in host: Probably abdominal cavity (found in wash).

Type locality: Off Nouméa, New Caledonia (collected 26 August 2010).

Prevalence and intensity: 1 of 7 fish examined infected; 1 nematode.

Deposition of type specimen (holotype mounted on SEM stub): Helminthological Collection of the Institute of Parasitology, Biology Centre of the Czech Academy of Sciences, České Budějovice, Czech Republic (Cat. No. N-1118).

Etymology: The specific name *dispar* (= unequal, disparate) is a Latin adjective and relates to the characteristic feature of this species, i.e. unequally long spicules.

### Description

*Male* (1 specimen, holotype): Body whitish, filiform, 5.14 mm long, maximum width at middle 63; anterior part of body slightly narrower just posterior to cephalic end (Figs. 5A and 6G); body width at this narrowed part 39. Maximum width/body length 1:82; width of cephalic end 45, that of posterior end 36. Cuticle smooth. Cephalic end rounded. Oral aperture small, oval, surrounded by 14 cephalic papillae arranged in two circles: external circle formed by four submedian pairs of papillae; internal circle formed by four submedian and two lateral papillae (Figs. 5B, 6A and 6B). Small lateral amphids just posterior to lateral papillae of internal circle in lateral views (Figs. 5B, 6A, 6B). Small deirids present at middle oesophageal region (Fig. 6F, 6G). Oesophagus 504 long, maximum width 27, forming 10% of body length, slightly inflated at anterior end; posterior part of muscular oesophagus overlapped by well-developed oesophageal gland with large cell nucleus situated somewhat posterior to its middle (Fig. 5A); anterior oesophageal inflation 24 long and 16 wide. Small ventriculus present. Nerve ring, excretory pore and oesophageal nucleus 183, 222 and 429, respectively, from anterior extremity. Testis reaching almost to posterior end of oesophagus (Fig. 5A). Posterior end of body blunt, forming distinct tail 9 long, with broad, U-shaped mound extending laterally (Figs. 5C, 6C–6E). Four pairs of very flat, hardly visible caudal papillae situated on sides of cloacal aperture and one pair of postanal papillae located more posteriorly on caudal mound (Figs. 5C, 6D and 6E). Pair of small phasmids present at about middle of each mound arm (Figs. 5C, 6D and 6E). Spicules slender, needle-like, very unequal, with somewhat expanded proximal and sharply pointed distal tips (Figs. 5E, 5F, 6C–6E); length of right spicule 123, comprising 2.3% of body length, that of left spicule 96; length ratio of spicules 1:1.28. Gubernaculum narrow, 102 long, with anterior portion slightly bent dorsally; length of anterior bent part 57, representing 56% of entire gubernaculum length; posterior portion of gubernaculum with distinct dorsal protuberance followed by small, reflexed barb located 27 from distal tip (Fig. 5D–5F). Length ratio of gubernaculum and longer (right) spicule 1:1.21. Spicules and gubernaculum well sclerotised; spicules brown-coloured, gubernaculum colourless.

*Female*: Not known.

### Remarks

To date, eight nominal species belonging to four philometrid genera (*Buckleyella* Rasheed, 1963, *Caranginema* Moravec, Montoya-Mendoza & Salgado-Maldonado, 2008, *Philometra* Costa, 1845 and *Philometroides* Yamaguti, 1935) are known to parasitise carangid fishes [42]. Four of these are known solely from their females, whereas males have been described only for *Caranginema americanum* Moravec, Montoya-Mendoza & Salgado-Maldonado, 2008 from subcutaneous tissues of *Caranx hippos* (L.) in the Gulf of Mexico, *Philometra austropacifica* Moravec & Justine, 2014 from the ovary of *Alepes vari* (Cuvier) off New Caledonia, *P*. *selaris* Moravec & Justine, 2014 from the abdominal cavity (?) of *Selar crumenophthalmus* (Bloch) off New Caledonia and *P*. *tauridica* Ivashkin, Kovaleva & Khromova in Ivashkin et al., 1971 from the abdominal cavity of *Trachurus mediterraneus* (Steindachner) in the Black Sea [21, 38, 42].

By the body length 5.1 mm, the present male resembles only that of *P*. *selaris* (5.3–5.5 mm), whereas the males of other three species are distinctly shorter (1.5–3.3 mm). Moreover, both *P*. *dispar* sp. n. and *P*. *selaris* possess a dorsal reflexed barb at the tip of the gubernaculum, which is absent in other species. However, the new species differs from *P*. *selaris* in having conspicuously unequal spicules (length ratio of spicules 1:1.28 *vs.* 1:1.03–1.04), a different shape and structure of the gubernaculum (presence *vs.* absence of a dorsal protuberance) and a more posterior location of the oesophageal cell nucleus; in addition, it was collected from a fish belonging to a different genus (*Carangoides vs. Selar*). Males of the remaining four philometrid species from carangids are not known and, consequently, cannot be compared with *P*. *dispar*; however, these species can be separated based on the different genus of their type host and their geographical distribution. The allocation of the new species to *Philometra* is provisional; present philometrid genera are mostly based on the morphology of gravid and subgravid females, whereas males of some genera (e.g. *Caranginema*, *Philometra* and *Philometroides*) are unidentifiable to genus [42].

By the very unequally long spicules, *P*. *dispar* n. sp. differs from all other philometrid species from marine fishes, for which males are known, except for *Philometra katsuwoni* Petter & Baudin-Laurencin, 1986 and *P*. *gymnosardae* Moravec, Lorber & Konečný, 2007, both gonad-infecting parasites of tuna fishes (Scombridae) in the Atlantic and Indian Oceans, respectively [16, 45, 55]. However, differences in the lengths of spicules of *P*. *katsuwoni* and *P*. *gymnosardae* are much more conspicuous as compared with that in *P*. *dispar*.

The authors are aware of the fact that this new species is being described from a single specimen, a procedure that cannot be generally recommended; however, in this case, the new species appears to be readily recognisable and, therefore, they consider it more reasonable and useful to give it a specific name rather than to report it only as *Philometra* sp.

## *Camallanus carangis* Olsen, 1954

Fam. Camallanidae Railliet et Henry, 1915

Syns.: *Camallanus marinus* Schmidt & Kuntz, 1969; *C*. *paracarangis* Velasquez, 1980.

Hosts: Longnose trevally *Carangoides chrysophrys* and *C*. *hedlandensis* (both Carangidae, Perciformes).

Site of infection: Digestive tract.

Localities: New Caledonia (JNC3172, collected 27 May 2010; JNC3212, 21 July 2010)

Prevalence and intensity: *C*. *chrysophrys*: 1 of 3 fish examined infected; 1 nematode. *C*. *hedlandensis*: 1/2; 1.

Deposition of voucher specimens: Muséum National d’Histoire Naturelle, Paris (JNC3172 (*C. hedlandensis*), JNC3212 (*C. chrysophrys*)).

### Remarks

Only two ovigerous female specimens, 11.06 mm and 18.43 mm long, were collected. The tail tip of both of them bears three small caudal projections. Since the general morphology of these specimens corresponds to that of *C*. *carangis*, as redescribed by Moravec et al. [44], they are assigned to this species.

*Camallanus carangis* was originally described by Olsen [49] from *Caranx* sp. in Fiji. At present, this species is known to occur in carangids and fishes belonging to some other families in Hawaii, French Polynesia, the Philippines and in the Arabian, Arafura, South China and Red Seas [27, 51, 60, 62, 69]. Moravec et al. [44] reported *C*. *carangis* from *Nemipterus furcosus* (Valenciennes) (Nemipteridae), *Parupeneus ciliatus* (Lacépède) and *Upeneus vittatus* (Forsskål) (both Mullidae) from off New Caledonia. The present findings of *C*. *carangis* in *C*. *chrysophrys* and *C*. *hedlandensis* represent new host records.

Moravec et al. [44] observed that the tail tip of young nongravid and small subgravid (ovigerous) females of *C*. *carangis* possess three small caudal projections, whereas these are totally absent from conspecific gravid (larvigerous) females. This is confirmed by the present study.

## *Johnstonmawsonia* sp. (Figs. 7, 8)

Fam. Rhabdochonidae Travassos, Artigas et Pereira, 1928

**Figure 7.**
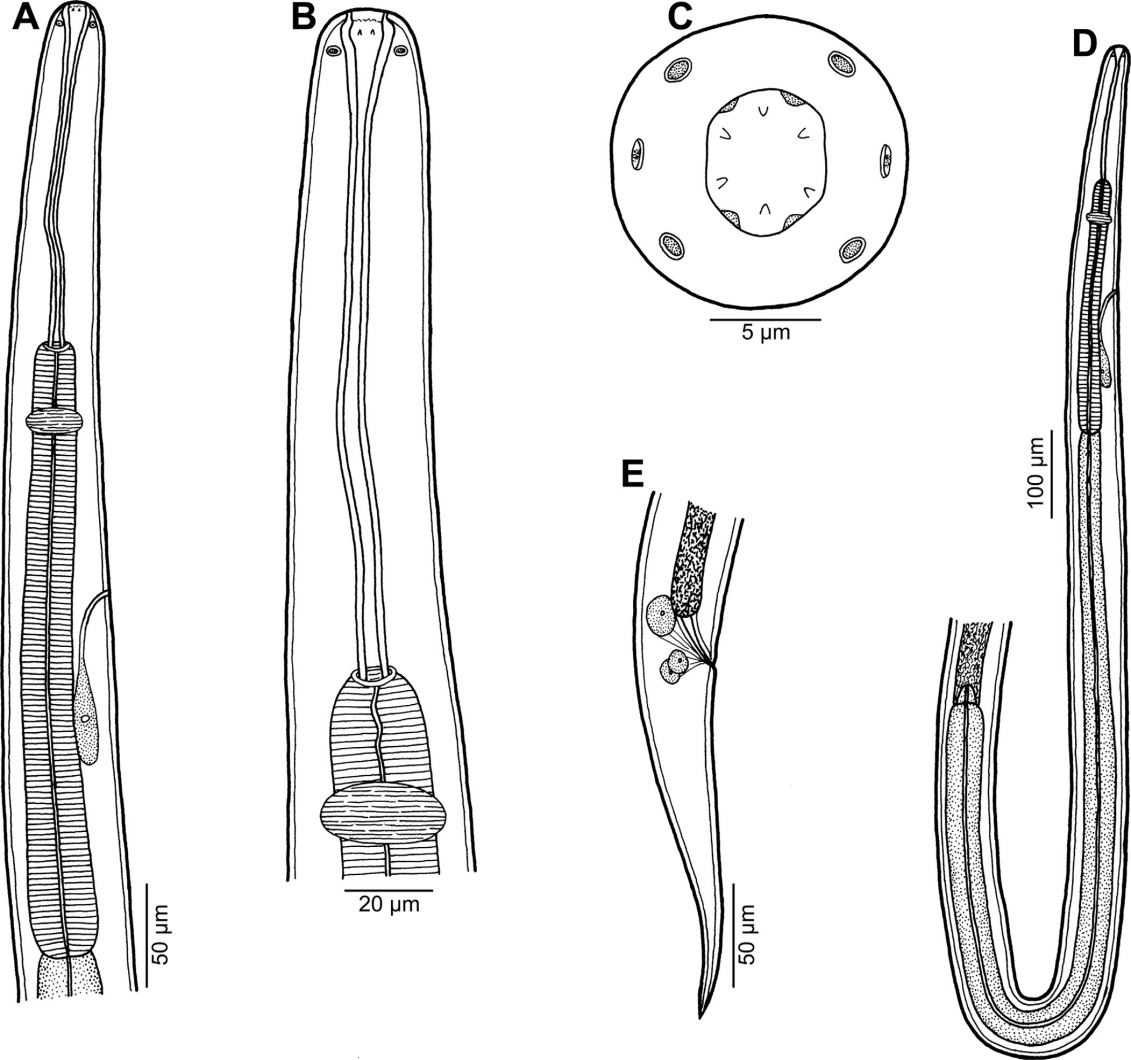
*Johnstonmawsonia* sp. from *Carangoides fulvoguttatus*, nongravid female. A: Anterior end of body, lateral view. B: Same, larger magnification. C: Cephalic end, apical view. D: Oesophageal portion of body, lateral view. E: Tail, lateral view.

**Figure 8.**
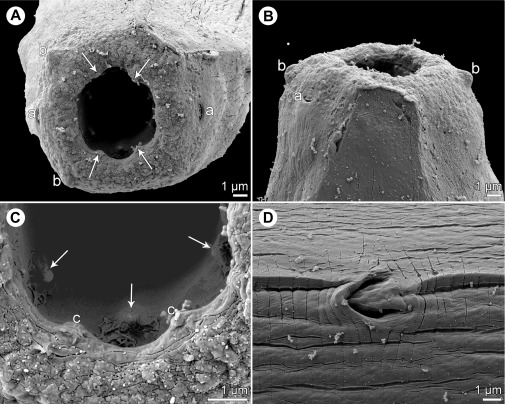
*Johnstonmawsonia* sp. from *Carangoides fulvoguttatus*, scanning electron micrographs of nongravid female. A, B: Cephalic end, apical and dorsoventral views, respectively (arrows indicate sublabia). C: Detail of mouth, apical view (arrows indicate inner prostomal teeth). D: Excretory pore, ventral view. *Abbreviations*: a, amphid; b, submedian cephalic papilla; c, sublabium.

Host: Yellowspotted trevally *Carangoides fulvoguttatus* (Carangidae, Perciformes); JNC3176 (see Table 1); Fork length 270 mm, weight 340 g.

Site in host: Probably abdominal cavity (found in wash).

Locality: Off Nouméa, New Caledonia (collected 28 May 2010).

Prevalence and intensity: 1 of 10 fish examined infected; 1 nematode.

Voucher specimen: Not maintained (used for SEM).

### Description

*Female* (1 nongravid specimen): Small, slender whitish nematode. Cuticle thin, finely transversely striated (Fig. 8D). Body 6.35 mm long, maximum width 69. Cephalic end rounded. Oral aperture hexagonal, surrounded by 4 submedian papillae and pair of lateral amphids (Figs. 7C, 8A, 8B). Pseudolabia absent. Four small submedian sublabia present (Figs. 7C, 8A, 8C). Deirids not found. Vestibule (stoma) long, with distinct funnel-shaped prostom at anterior end (Fig. 7A, 7B and 7D); prostom without anterior, subterminal teeth, but with 6 (1 dorsal, 1 ventral and 2 lateral on either side) minute denticles extending anteriorly from inner wall of prostom more posteriorly (Figs. 7A–7C, 8A and 8C); length of vestibule including prostom 171; prostom 24 long, 15 wide. Muscular oesophagus 318 long, maximum width 33, somewhat expanded towards its posterior end, well separated from glandular oesophagus; glandular oesophagus 1.13 mm long, maximum width 45, opens into intestine through large valve; length ratio of both oesophageal portions 1:3.55 (Figs. 7A, 7B and 7D). Length of vestibule and entire oesophagus represents 25% of body length. Intestine narrow, pale-coloured. Nerve ring encircles muscular oesophagus approximately at its first seventh; excretory pore located somewhat anterior to mid-length of muscular oesophagus (Figs. 7A, 7D and 8D). Nerve ring and excretory pore 213 and 300, respectively, from anterior extremity. Vulva situated 3.60 mm from anterior end of body, i.e. at 57% of body length. Vagina short. Uterus little developed, empty, formed by narrow tube provided with reflexed ovaries at both ends. Three distinct unicellular rectal glands present dorsally from rectum. Tail conical, 183 long, with pointed tip (Fig. 7E).

### Remarks

In having a hexagonal oral aperture, no pseudolabia and a long vestibule (stoma) forming a distinct funnel-shaped prostom at its anterior end, the single available female specimen evidently belongs to the thelazioid family Rhabdochonidae. At present, this family consists of 11 genera, of which *Trichospirura* Smith & Chitwood, 1967 contains several species parasitising tetrapod vertebrates (amphibians, reptiles and mammals) [2], whereas the nematodes belonging to all other genera are parasites of fishes [36].

Of these, representatives of three genera, *Rhabdochona* Railliet, 1916, *Prosungulonema* Roytman, 1963 and *Beaninema* Caspeta-Mandujano, Moravec & Salgado-Maldonado, 2001, are parasites of freshwater fishes; whereas *Rhabdochona* contains more than 100 species (all intestinal parasites) distributed in all main zoogeographical regions [37], *Prosungulonema* and *Beaninema* are represented by a few species parasitic in the host’s intestine, liver, gall-bladder or swimbladder in eastern Asia, Africa and Mexico [17, 36]. On the contrary, the remaining seven genera, *Johnstonmawsonia* Campana-Rouget, 1955, *Hepatinema* Rasheed, 1964, *Heptochona* Rasheed, 1965, *Vasorhabdochona* Martin & Zam, 1967, *Pancreatonema* McVicar & Gibson, 1975, *Fellicola* Petter & Køie, 1993, and *Megachona* Mejía-Madrid & Pérez-Ponce de León, 2007, contain only several species (most of these genera are monotypic) that are parasites in the digestive tract and associated glands, bloodstream and body cavity of marine and brackish-water fishes in tropical and subtropical regions [3, 33, 45, 47].

In having the nerve ring encircling the muscular oesophagus, a well-developed funnel-shaped prostom and no anterior prostomal teeth, the available nematode specimen from *C*. *fulvoguttatus* distinctly differs from species of *Beaninema*, *Fellicola*, *Hepatinema*, *Heptochona*, *Pancreatonema*, *Prosungulonema*, *Rhabdochona*, *Megachona* and *Trichospirura* [36]. From the monotypic *Vasorhabdochona*, it can be differentiated by the didelphic (*vs.* monodelphic) female genital tract and an almost equatorial location of the vulva (at 53% *vs.* 8–19% of body length in *Vasorhabdochona*) [20, 32, 47]. Therefore, the present New Caledonian specimen is provisionally assigned to *Johnstonmawsonia*.

At present, the genus *Johnstonmawsonia* is represented by the following four species: *J*. *campanarougetae* Machkovskiy & Parukhin, 1979, *J*. *coelorhynchi* (Johnston & Mawson, 1945) (type species), *J*. *muraenophidis* Campana-Rouget, 1955, and *J*. *porichthydis* Tanzola & Gigola, 2002, all parasites of the digestive tract and pancreatic ducts of marine fishes [14, 22, 30, 68]. Two additional species of *Johnstonmawsonia* were described from freshwater fishes in Africa [46, 57], but, with respect to the papers by Roytman and Ivanova [61] and Moravec et al. [47], these should be transferred to *Prosungulonema* as *P*. *africanum* (Moravec & Puylaert, 1970) n. comb. and *P*. *campanae* (Puylaert, 1973) n. comb.

All species of *Johnstonmawsonia* were described to have no teeth in the prostom. The present specimen has no anterior prostomal teeth, but its prostom is provided with six minute, more posteriorly located denticles, which are visible only with the use of SEM. However, it should be remarked that none of the *Johnstonmawsonia* spp. has so far been studied by SEM (except for the poor quality SEM micrograph of the cephalic end of *J*. *porichthydis* [68]). Therefore, it is not currently clear whether the presence of small posterior prostomal denticles is a generic feature in *Johnstonmawsonia* or it is only a character of an apparently undescribed congeneric species parasitising *Carangoides fulvoguttatus*.

The presence of posteriorly located prostomal teeth, as found in the present specimen of *Johnstonmawsonia*, is not exceptional among rhabdochonids. *Megachona chamelensis* Mejía-Madrid & Pérez-Ponce de León, 2007, a parasite of intestinal caecae of *Kyphosus ocyurus* (Jordan & Gilbert) (Kyphosidae) off the Pacific coast of Mexico, possesses many large anterior prostomal teeth and numerous smaller teeth with an irregular arrangement located more posteriorly [33]. On the other hand, the anterior prostomal teeth are absent in *Fellicola longispiculus* Petter & Køie, 1993, a parasite of the gall-bladder of *Coryphaenoides rupestris* Gunnerus (Macrouridae) from off the Faroes, North Atlantic, but its prostom is internally lined with six longitudinal thickenings (four lateral simple and two median bilobed) appearing anteriorly as small denticles [56]. In *Beaninema nayaritense* Caspeta-Mandujano, Moravec & Salgado-Maldonado, 2001, a parasite of the gall-bladder of *Cichlasoma beani* (Jordan) (Cichlidae) in Mexico, the anterior prostomal teeth are absent, but there are six large conical teeth in the posterior half of the prostom [17]. Both *B*. *nayaritense* and *F*. *longispiculus* markedly differ from *Johnstonmawsonia* spp. in having the nerve ring that encircles the vestibule instead of the muscular oesophagus.

Since no rhabdochonid nematodes were previously recorded from carangid hosts, it is almost certain that the recorded specimen of *Johnstonmawsonia* sp. from *C*. *fulvoguttatus* belongs to an undescribed species. However, because only a single nongravid female was available, we refrain from describing this new taxon.

### Conflict of Interest

The Editor-in-Chief of Parasite is one of the authors of this manuscript. COPE (the Committee on Publication Ethics, http://publicationethics.org), to which Parasite adheres, advises special treatment in these cases. In this case, the peer review process was handled by an Invited Editor, Jérôme Depaquit.
